# The role of bacterial signaling networks in antibiotics response and resistance regulation

**DOI:** 10.1007/s42995-022-00126-1

**Published:** 2022-03-28

**Authors:** Yuying Li, Tao Feng, Yan Wang

**Affiliations:** 1 College of Marine Life Sciences, Ocean University of China, Qingdao, 266003 China; 2 Institute of Evolution and Marine Biodiversity, Ocean University of China, Qingdao, 266003 China; 3 Laboratory for Marine Ecology and Environmental Science, National Laboratory for Marine Science and Technology (Qingdao), Qingdao, 266071 China

**Keywords:** Bacterial antibiotic resistance, AHL, AI-2, AIP, Indole, DSF

## Abstract

Excessive use of antibiotics poses a threat to public health and the environment. In ecosystems, such as the marine environment, antibiotic contamination has led to an increase in bacterial resistance. Therefore, the study of bacterial response to antibiotics and the regulation of resistance formation have become an important research field. Traditionally, the processes related to antibiotic responses and resistance regulation have mainly included the activation of efflux pumps, mutation of antibiotic targets, production of biofilms, and production of inactivated or passivation enzymes. In recent years, studies have shown that bacterial signaling networks can affect antibiotic responses and resistance regulation. Signaling systems mostly alter resistance by regulating biofilms, efflux pumps, and mobile genetic elements. Here we provide an overview of how bacterial intraspecific and interspecific signaling networks affect the response to environmental antibiotics. In doing so, this review provides theoretical support for inhibiting bacterial antibiotic resistance and alleviating health and ecological problems caused by antibiotic contamination.

## Introduction

Antibiotics are being introduced to numerous environments, leading to new or enhanced antibiotic resistance by bacteria. This resistance then poses a threat, potentially leading to bacterial contamination and ecological imbalance. Over the last 20 years, numerous antibiotic-resistant bacteria have been isolated from the marine environment, especially in coastal waters with severe antibiotic pollution (Matyar 2012; Sundaramanickam et al. 2015). There is, therefore, a need to understand the mechanism leading to resistance, which is a purpose of this review.

The impact on sea turtles is a good example of the consequences of antibiotic resistance in marine environments. In fact, sea turtles are considered sentinels for detecting antibiotic-resistant bacteria. For instance, Al-Bahry et al. (2011) sampled oviductal fluid from 40 sea turtles in Ras Al-Hadd, Oman, and ~ 60% of 132 isolated bacterial strains showed multiple resistance to antibiotics. Likewise, Trotta et al. (2021) isolated 40 Gram-negative bacteria from the wounds of 52 injured sea turtles living in the Mediterranean, and found that 75% of them were multidrug-resistant bacteria. These data highlight why understanding antibiotic resistance in marine bacteria is needed.

**Fig. 1:**
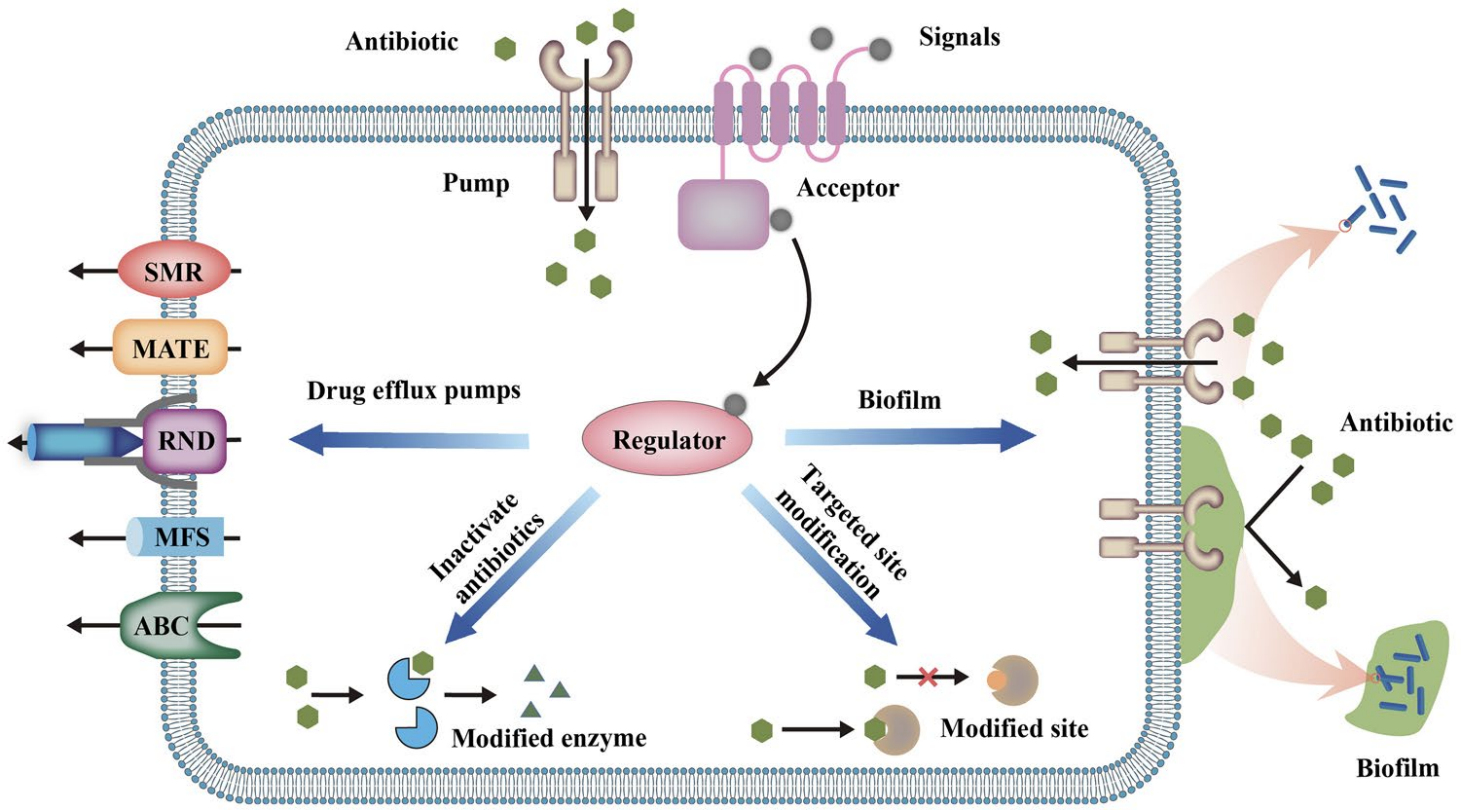
Mechanisms of bacterial antibiotic resistance

Although our review does not specifically focus on marine bacteria, all the processes described below are directly relevant to marine systems. For the sake of brevity, however, we have restricted our analysis to examine general processes. It will be the work of future studies to apply them to marine bacteria. The known mechanisms of antibiotic response and bacterial resistance mainly include the overexpression of efflux pumps, modification of antibiotic-targeted sites, production of antibiotic-modifying enzymes, and formation of biofilms (Stewart 2002; Wright 2011; Zgurskaya 2002) (Fig. 1). In recent years, studies have reported a strong correlation between bacterial signaling systems and bacterial resistance (Wang et al. 2019a; Wu et al. 2020; Zhao et al. 2020). Correspondingly, several studies suggest a novel strategy to reverse antimicrobial resistance through regulating bacterial signaling networks. For example, Rezzoagli et al. (2020) found that in the combined use of furanone C-30, an inhibitor of the *las* system, and tobramycin, furanone C-30 can completely restore the inhibition of bacterial growth by antibiotics. In this review, we will examine how signaling networks regulate antibiotic resistance and analyze the feasibility of various signaling systems to participate in the inhibition of bacterial resistance. The main signaling systems included in this review are: (1) the *N*-acyl-homoserine lactone (AHL) system in Gram-negative bacteria; (2) the autoinducer-2 (AI-2) system in both Gram-negative and Gram-positive bacteria; (3) the auto-inducing peptide (AIP) system in Gram-positive bacteria; and (4) bacterial communication systems involving other signaling molecules, such as indole, diffusible signal factor (DSF), cyclic di-guanosine monophosphate (c-di-GMP), *Pseudomonas* quinolone signal (PQS), and autoinducer-3 (AI-3) (Table 1). Each of these topics is examined below.

**Table 1:** Types of bacterial signaling systems

QS signaling molecule type	Representative bacteria	Selected phenotype
AHLs (Ng and Bassler 2009; Taguchi et al. 2006)	Various Gram-negative bacteria, such as *Vibrio* spp. and *Pseudomonas aeruginosa*	Virulence, biofilm formation, swarming and bioluminescence
AI-2 (Pereira et al. 2013)	Many Gram-negative and Gram-positive bacteria such as *Vibrio harveyi* and *Streptococcus bovis*	Virulence, biofilm formation, antibiotic synthesis
Indole (Lee et al. 2015)	Many Gram-negative and Gram-positive bacteria, such as *Escherichia coli* and *Symbiobacterium thermophilum*	Virulence, biofilm formation
DSF/BDSF (Poplawsky et al. 2005)	*Xanthomonas* spp. and *Burkholderia cenocepacia*	Virulence, biofilm formation
AIPs (Saenz et al. 2000)	Many Gram-positive bacteria, such as *Streptococcus pneumonia*	Virulence, biofilm formation, exopolysaccharide
PQS/IQS (Diggle et al. 2006; Lee et al. 2013)	*Pseudomonas aeruginosa*	Virulence and biofilm formation, extracellular polysaccharide
AI-3 (Moreira and Sperandio 2016)	Enterohemorrhagic *Escherichia coli* (EHEC)	Virulence
DKPs (Lee 2008)	*Pseudomonas aeruginosa*	Virulence and biofilm formation
CAI-1 (Kelly et al. 2009)	*Vibrio* spp. and *Legionella pneumophila*	Virulence and biofilm
Pyrones (Brachmann et al. 2013)	*Photorhabdus luminescens*	Virulence
DARs (Brameyer et al. 2015)	*Photorhabdus asymbiotica*	Virulence
CHDs (Brameyer et al. 2015)	*Photorhabdus asymbiotica*	Virulence

*AHL* *N*-acyl-homoserine lactone, *AI-2* autoinducer-2, *DSF* diffusible signal factor, *BDSF* *Burkholderia cenocepacia* diffusible signal factor, *AIP* autoinducing peptide, *PQS* *Pseudomonas* quinolone signal, *IQS* integrating quorum sensing signal, *AI-3* autoinducter-3, *DKPs* diketopiperazines, *CAI-1* *Cholerae* autoinducer-1, *DARs* dialkylresorcinols, *CHDs* cyclohexanediones

## *N*-acyl-homoserine lactone (AHL)

**Fig. 2:**
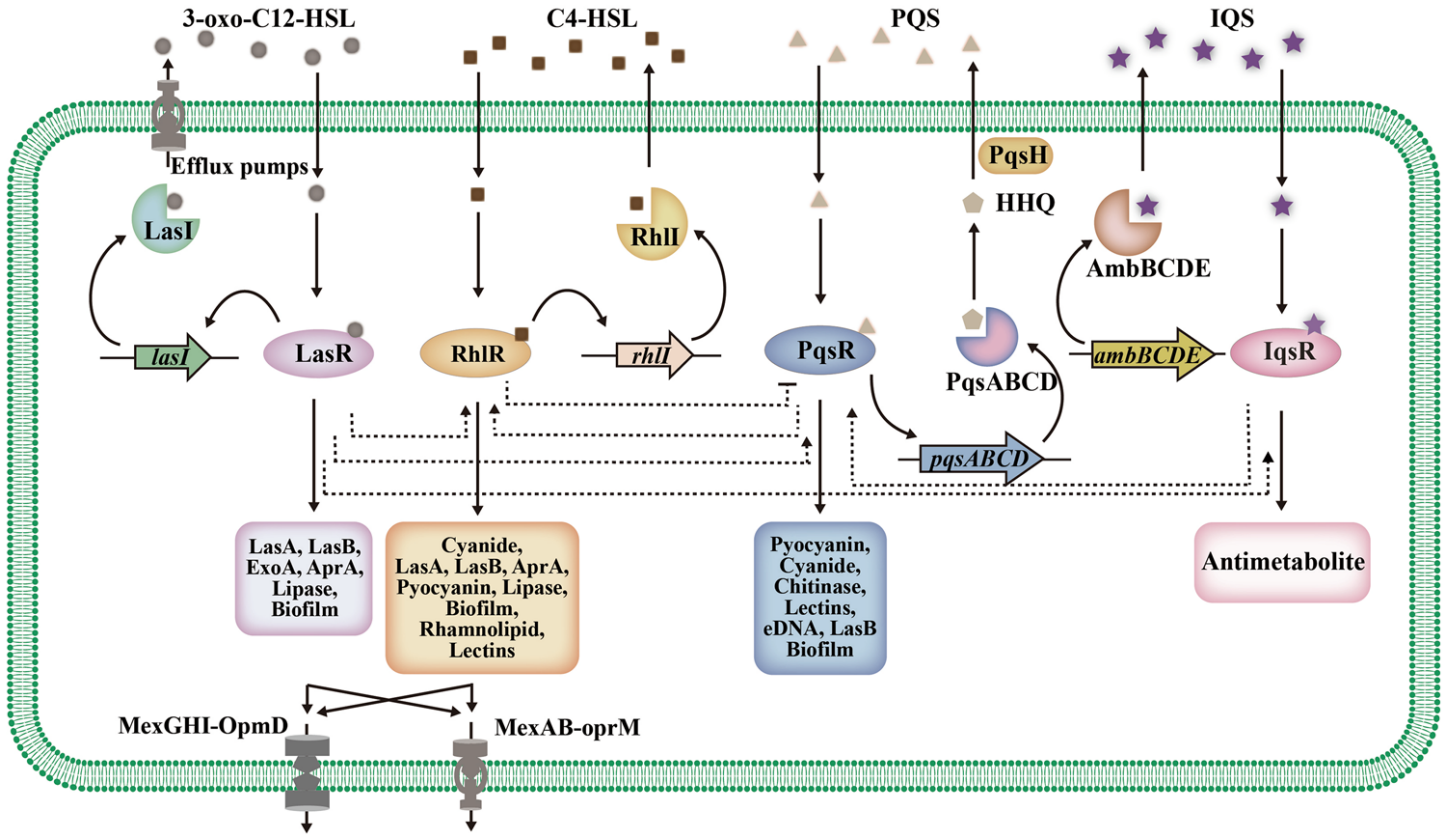
The AHL-mediated signaling system. There are four signaling molecular regulatory pathways in *Pseudomonas aeruginosa*: the Las system, regulated by *lasI* and *lasR* genes; the Rhl system, regulated by *rhlR* and *rhlI* genes; the PQS system (which can also be regulated by LasR); and the IQS system. The solid arrows represent the signal transmission process, and the dashed arrows show the interrelationship between different signaling molecular regulatory pathways. These signaling molecular regulatory pathways interact with each other, forming a complex regulatory network to regulate the biosynthesis of biofilms and the expression of virulence factors and efflux pumps (Chatterjee et al. 2016; Diggle et al. 2006; Lee et al. 2013)

AHL signaling molecules are the most common and well-studied auto-inducers in Gram-negative bacteria. Different bacteria produce different AHL signaling molecules, but they all work in a similar manner, and the distinctions between them are mainly the length of the amide side chain and the substituent group (hydrogen group, hydroxyl group or carbonyl group) on the third carbon atom (Fuqua et al. 2001). Examples of AHL signaling systems include the LasI/R system and the RhlI/R system in *Pseudomonas aeruginosa* (Fig. 2) (Chatterjee et al. 2016), the CepI/R system in *Burkholderia cenocepacia* (Subramoni and Sokol 2012), the CviI/R system in *Chromobacterium violaceum* (de Oca-Mejia et al. 2015), and the AhyI/R system in *Aeromonas hydrophila* (Jahid et al. 2015).

The mechanism by which the AHL signaling system affects bacterial antibiotic resistance is complex, with the two key factors being biofilms and efflux pumps. *P. aeruginosa*, a common opportunistic pathogen with multidrug resistance and the most studied bacterium of the AHL system, has two known AHL signaling systems, LasI/R and RhlI/R. LasI/R system can synthesize *N*-(3-oxododecanoyl)-l-homoserine lactone (3-oxo-C12-HSL) by LasI and recognize it by LasR, while RhlI/R system can synthesize and recognize *N*-butyryl-l-homoserine lactone (C4-HSL) by RhlI and RhlR (Chatterjee et al. 2016). Both of them have an effect on biofilms and efflux pumps in *P. aeruginosa*. The LasI/R system determines the structure of bacterial biofilms and plays a crucial role in bacterial adhesion, while the RhlI/R system maintains the basic structure of biofilms by regulating the production of rhamnolipids (Kjelleberg and Molin 2002; Mattmann et al. 2008). The interference of biofilm formation is often accompanied by the increase of antibiotic sensitivity (Yu et al. 2015). Zhao et al. (2015) have demonstrated that the AHL system could lead to a higher level of resistance by increasing biofilm formation and antimicrobial-induced *ampC* expression. In addition, 3-oxo-C12-HSL and C4-HSL can simultaneously induce the expression of the *qsc133* gene cluster, which can express the novel RND efflux pump MexGHI-OpmD (Aendekerk et al. 2002). At the same time, C4-HSL induces expression of the *mexAB-oprM* efflux pump gene and enhances resistance to chloramphenicol, fluoroquinolones and most *β*-lactam antibiotics (Maseda et al. 2004; Sawada et al. 2004).

Bacterial pathogenicity can be effectively weakened and antibiotic resistance may be delayed by inhibiting signaling networks (LaSarre and Federle 2013). There are several ways to inhibit AHL signaling system. The most common approach is to interfere with the synthesis of AHL signals by inhibiting AHL synthetases. LuxI synthetases play an important role in the synthesis of AHL signaling molecules and are conserved in hundreds of Gram-negative bacteria, making them an ideal target for signaling inhibitors (Chan et al. 2015). Lidor et al. (2015) found that the binding of (*z*)-5-octylidenethiazolidine-2,4-dione (TZD-C8) to the active site of LasI synthase interfered with the synthesis of 3-oxo-C12-HSL signaling molecules and inhibited the biofilm formation of *P. aeruginosa* PAO1 in a dose-dependent manner. Similarly, in the Gram-negative bacterium *Burkholderia glumae*, the inhibitor J8-C8 bound to the acyl-ACP-binding site on the synthase TofI, inhibiting the production of C8-HSL by competing with substrates (Chung et al. 2011). In addition to inhibiting AHL synthetases directly, the synthesis of signals might be blocked by inhibiting the precursor synthesis of AHL. For example, triclosan can inhibit the acyl carrier protein reductase (FabI) of *P. aeruginosa* to decrease the level of butyryl-ACP, which is necessary for the synthesis of C4-HSL signals by RhlI synthase (Hoang and Schweizer 1999). However, the inhibition of substrate synthesis may affect fatty acid metabolism and interfere with the crucial life activities of bacteria, causing the bacteria to develop new resistance. In fact, in some studies, resistance to triclosan has been reported (Chuanchuen et al. 2003).

Second, the use of AHL lactonase and AHL acylase enzymes, which can degrade AHL signals, is a theoretically feasible way to inhibit communication. AHL lactonase is a broad-spectrum enzyme because of its ability to hydrolyze the conserved HSL ring of AHL signals (Dong and Zhang 2005). Almost all AHL lactonases are metallo-*β*-lactamases, such as AiiA and AttM. They have the highly conserved Zn^2+^-binding domain HXHXDH-60aa-H, which is necessary for AHL lactonase activity (Thomas et al. 2005). Recently, a novel ocean-derived AHL lactonase, MomL, which has a broad substrate range and can effectively inhibit the expression of pathogenic factors and the formation of biofilms of pathogenic bacteria, was reported to effectively degrade AHL signals, and the efficiency of C6-HSL degradation was as high as 2.9 × 10^5^ s^−1^ M^−1^ (Tang et al. 2015). In terms of removing auto-inducers and biofilms and improving the sensitivity of *P. aeruginosa* to antibiotics, MomL exhibited much better catalytic efficiency compared with other AHL lactonases (Sedlmayer et al. 2017)*.* Finally, interfering with the binding of signaling molecules to receptors is another mode of action for inhibitors. Furanones produced by *Delisea pulchra* can inhibit the signaling communication of bacteria, greatly reducing the resistance of biofilms to antibiotics (Hentzer et al. 2003). The structures of furanones are similar to those of HSLs, so they can inhibit the AHL signaling system of *Vibrio harveyi*, a pathogen of fish and invertebrates (Zhang et al. 2020), by binding to the receptor protein LuxR in competition with AHL signaling molecules (Defoirdt et al. 2007). Ajoene in garlic extract and patulin in *Penicillium* inhibit the AHL signaling system of *P. aeruginosa* by inhibiting the binding of receptors to AHL signals, increasing the sensitivity to tobramycin (Jakobsen et al. 2012).

In addition to the selective pressure posed by the natural and host environments, antibiotic resistance can also be provided by antibiotic resistance genes located on mobile genetic elements (Ma et al. 2014). In future, it is hoped that the bacterial signaling system will address the resistance that arises under selection pressure. However, what is even more surprising is that the conjugative transfer of resistance genes is also expected to be solved by signaling systems. Studies show that AHLs secreted by bacteria can promote conjugative transfer of antibiotic resistance genes in the environment by enhancing mRNA expression (Zhu et al. 2020). The conjugative transfer frequency was decreased by AHL inhibitors (furanone, benzpyrole, coumarin) and decreased with increasing inhibitor concentration (Zhu et al. 2020).

Obviously, AHL signaling molecules strengthen the antibiotic resistance of bacteria, such as *P. aeruginosa*. AHLs have relatively well-conserved synthetic gene sequences, and their molecular structures and action mechanisms are very similar (Fuqua et al. 2001), so investigating model bacteria to find an approach to suppress bacterial resistance by inhibiting the AHL signaling system is viable. Enzymes possessing an ability to degrade AHL are an ideal choice for signaling inhibitors. AHL does not inhibit the growth of bacteria and consequently has no effect on antibiotic resistance. Kordbacheh et al. (2017) found a competitive inhibitor, *Pistacia atlantica* methanolic leaf extract. Its high affinity for LasR protein can prevent communication signals from being transmitted downward and inhibit the AHL signaling pathway. In addition, the minimum biofilm inhibitory concentration (MBIC) of *P. atlantica* crude extract against *P. aeruginosa* biofilm was only 0.25 mg/ml, and the inhibitory rate was 39%, indicating that the crude extract has the potential to treat chronic infection caused by *P. aeruginosa*.

## Autoinducer-2 (AI-2)

**Fig. 3:**
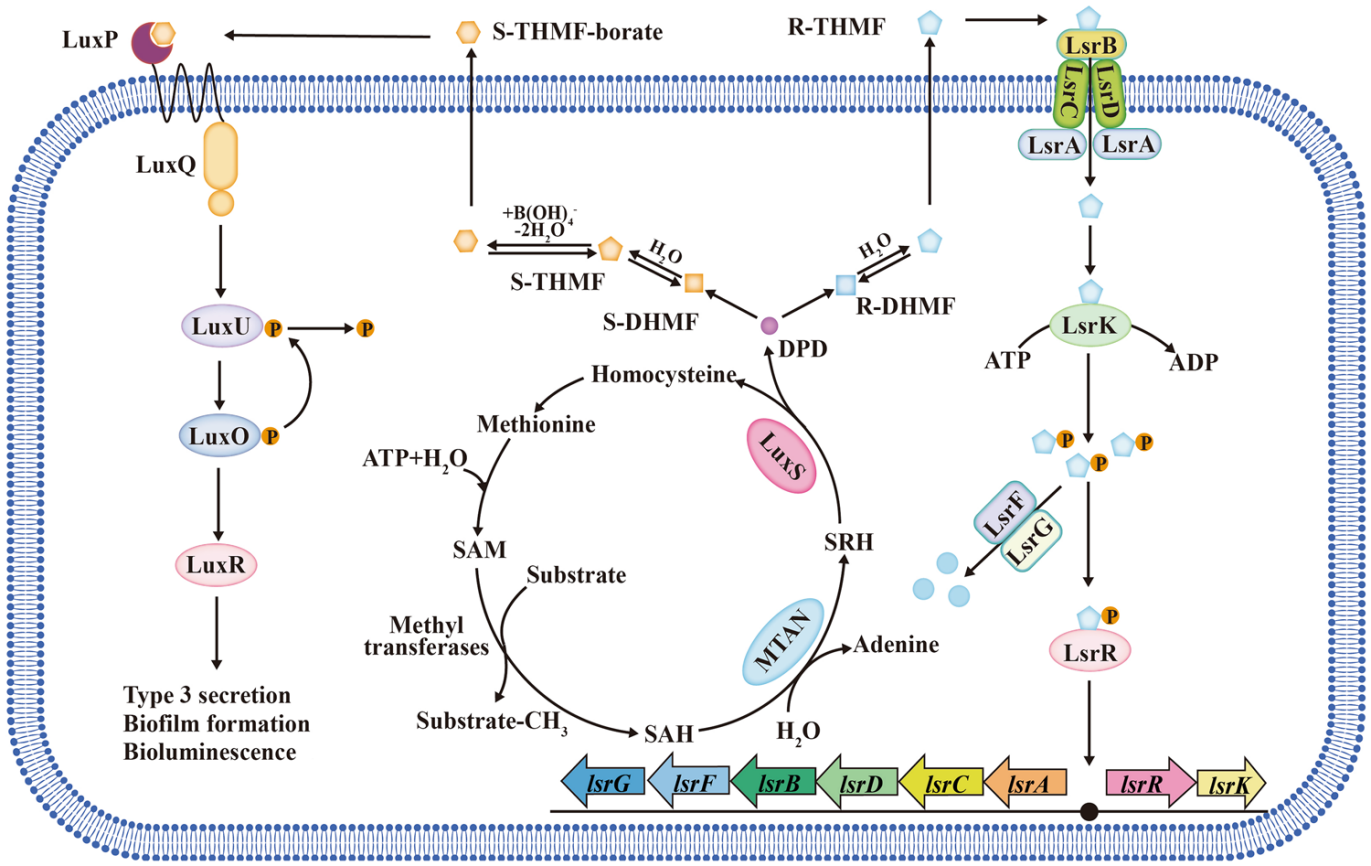
The AI-2-mediated signaling system. The synthesis of AI-2 is linked to the metabolism of S-adenosylmethionine (SAM) in the activated methyl cycle (AMC) (Song et al. 2018). The structure of 4,5-dihydroxy-2,3-pentanedione (DPD), a precursor molecule of AI-2, is unstable and easily forms different isomers in different bacteria (Vendeville et al. 2005). In *V. harveyi*, the isomers of the AI-2 signaling molecule are borated (2S,4S)-2-methyl-2,3,3,4-tetrahydroxytetrahydrofuran-borate (S-THMF-borate) (Chen et al. 2002). However, the AI-2 signaling molecule of *Salmonella trophimurium* is nonborated (2R,4S)-2-methyl-2,3,3,4-tetrahydroxytetrahydrofuran (R-THMF) (Miller et al. 2004)

The AI-2-mediated signaling system (Fig. 3), also called the LuxS/AI-2 molecular signaling system (Surette et al. 1999), is widely present in bacteria and regulates many important physiological processes, such as toxin secretion, antibiotic synthesis, biofilm formation, and antibiotic resistance (Guo et al. 2018; Vidal et al. 2015; Yadav et al. 2018). The AI-2 signaling molecule produced by the *luxS* gene in some bacteria is recognized by different bacteria species and plays an important role in transformation, so it is considered a signaling molecule for interspecific communication. The *luxS* gene and its synthetic product, the signaling molecule AI-2, play an essential role in bacterial resistance (Wang et al. 2019a). Kaur et al. (2020) recently revealed that heterogeneous expression of LuxS of *Meiothermus ruber* in *E. coli* increased the sensitivity to antibiotics, and the MICs of antibiotics, such as kanamycin, gentamycin, and chloramphenicol, were reduced by half or more. In addition, other studies on AI-2 and bacterial resistance have emerged recently (Linciano et al. 2020). Therefore, it is theoretically feasible to regulate bacterial resistance through the AI-2 system.

AI-2 can regulate antibiotic resistance because of its regulation of biofilm formation by various bacteria (Kaur et al. 2020; Zhang et al. 2019). The *luxS* gene can not only directly affect the synthesis and structure of biofilms but also indirectly affect the structure of biofilms through physiological metabolism, while overexpression or exogenous addition of AI-2 can lead to an increase in biofilm biomass (Sun et al. 2014). For example, Xue et al. (2015) found that the exogenous addition of AI-2 can increase the transcription of the *ica* and *bhp* genes encoding biofilm-related proteins and increase the biofilm biomass in *Staphylococcus epidermidis*. In addition to biofilms, the AI-2/LuxS system can regulate bacterial resistance by affecting antibiotic efflux pumps. The binding of AI-2 to a receptor causes overexpression of the *satAB* efflux pump gene, resulting in bacterial resistance, but deletion of the *luxS* gene inhibited the expression of the *satAB* efflux pump gene and enhanced the sensitivity of *Streptococcus suis* to quinolone antibiotics (Wang et al. 2019b). The MICs of norfloxacin and enrofloxacin for the *luxS* deletion strain of *S. suis* were half of that in the corresponding wild-type strain (2.5 vs. 5 mg/L, 0.625 vs. 1.25 mg/L); exogenous AI-2 restored the MICs of the *luxS* deletion strain to a normal value and doubled the value of the wild-type *S. suis* strain. Finally, the AI-2/LuxS system can also affect resistance conferred by mobile genetic elements (Xue et al. 2016). Exogenous AI-2 influenced the tetracycline resistance of *S. suis* by upregulating the expression of the antibiotic resistance gene *tet (M)* located in the transposon Tn916 family (Liu et al. 2020). Therefore, regulating the expression of bacterial efflux pumps by inhibiting the AI-2/LuxS system is expected to overcome antibiotic resistance.

The inhibitor MT-DADMe-ImmA can inhibit the AI-2/LuxS system of *Vibrio cholerae* without affecting growth rate by inhibiting S-adenosylhomocysteine nucleosidase (MTAN), which is an intermediate enzyme for the synthesis of AI-2 signals (Singh et al. 2005). MTAN can degrade S-adenosylhomocysteine (SAH) to form the substrate S-ribosylhomocysteine (SRH) of LuxS synthase, and then LuxS promotes the synthesis of 4,5-dihydroxy-2,3-pentanedione (DPD), a precursor of AI-2 signaling molecules (Fig. 3). MTAN is a good target for signaling inhibitors because it is found only in bacteria, similar to LuxS synthase. Inhibition of MTAN leads to the accumulation of methylthioadenosine (MTA) and SAH and inhibits the production of AI-2 (Schramm et al. 2008; Singh et al. 2005). However, further studies found that excessive MTA and SAH affect cell growth and DNA synthesis (Heurlier et al. 2009; Yang et al. 2016), so an inhibitor of MTAN may cause new resistance. In contrast, inhibitors of LuxS synthase, which is not necessary for bacterial survival, are more practical. The small peptides 5411 and 5906 can strongly bind LuxS to affect AI-2 production by *Edwardsiella tarda*, thereby inhibiting the expression of virulence factors and biofilm formation (Zhang et al. 2009).

Inactivation or denaturation of AI-2 signals can inhibit the AI-2/LuxS system and prevent bacterial resistance. This strategy does not directly kill the bacteria by inhibiting vital target genes, so new resistance rarely emerges. The use of LsrK, a key kinase in the AI-2/LuxS system that can phosphorylate AI-2 transported into cells, is a breakthrough in this strategy. Roy et al. (2010) found that the signaling communication responses in *Escherichia coli*, *Salmonella typhimurium* and *Vibrio harveyi* were significantly attenuated when exogenous ATP and LsrK were added. The addition of these substances causes AI-2 signals to be phosphorylated outside the cell, and phosphorylated AI-2 signals cannot normally pass through the cell membrane and play a regulatory role (Zhu et al. 2013). Similarly, endogenous LsrK deficiency leads to the extracellular accumulation of AI-2 signals because phospho-AI-2 cannot be produced (Xavier and Bassler 2005). Moreover, since LsrK can also phosphorylate DPD (Fig. 3), the highly active precursor of AI-2, a strategy of inhibiting LsrK may be effective for the AI-2/LuxS system in various bacteria, regardless of the differences in AI-2 signal structure and regulatory mechanism (Xavier et al. 2007). As a result, Linciano et al. (2020) carried out a comprehensive analysis of the design of LsrK inhibitors and recognized their potential in combating antimicrobial resistance.

In the LuxS/AI-2 system, AI-2 signals play a regulatory role in controlling the expression of target genes after binding to receptor proteins, so destruction of signal recognition and transduction can also play an inhibitory role. The compound pyrogallol and its analogs can suppress the signaling communication of *V. harveyi* by inhibiting the activity of the receptor protein LuxP (Ni et al. 2008). Peng et al. (2009) also found that two synthetic compounds, KM-03009 and SPB-02229, combined with LuxP to inhibit the AI-2/LuxS system of *V. harveyi*. In addition, furanone and cinnamaldehyde from natural sources were found to suppress the signaling pathway by inhibiting the binding of the response regulator LuxR to target genes (Brackman et al. 2011; Defoirdt et al. 2007). Rhoads et al. (2017) assembled a functionalized biopolymer capsule attached to LsrK. It can regulate AI-2-mediated signaling system and has the potential to be applied to human wound dressings to prevent infection. This capsule blocks signaling communication, which could help slow the development of antibiotic resistance caused by the AI-2 system and provide a new strategy for material development that blocks the signaling systems.

## Autoinducer peptides (AIPs)

The AIP-mediated signaling system is one of the main communication modes of Gram-positive bacteria. AIP signals usually consist of 5–17 amino acids with side chains that generally contain modifying groups, such as isopentene and thiolactone rings (Murray and Williams 2018). During growth, Gram-positive bacteria encode precursor peptides that can be processed and modified to form stable and active AIP signals. AIP signals secreted by different bacteria vary in size and cannot pass through the bacterial cell wall by free diffusion. Therefore, the assistance of membrane channel proteins or ABC transport systems is needed to transport AIP signals outside the cell to perform functions (Haque and Yadav 2019).

**Fig. 4:**
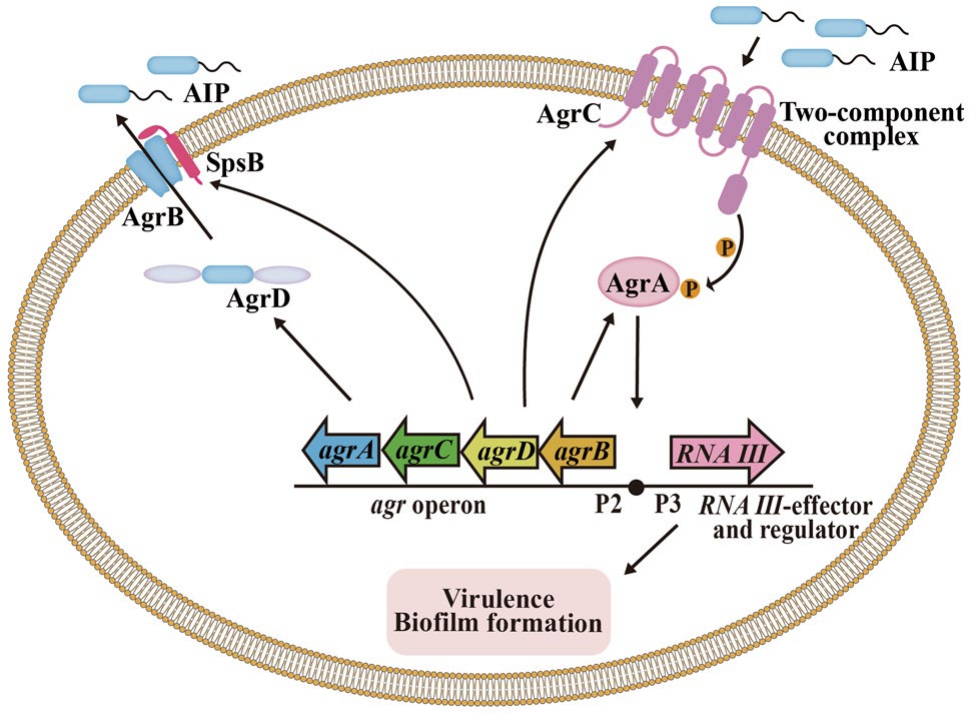
The AIP-mediated signaling system

Taking the *agr* signaling system of *Staphylococcus aureus* as an example to introduce the regulation mode of the AIP signaling system (Fig. 4), the pre-AIP encoded by *agrD* is first processed to a mature AIP by the AgrB endopeptidase and the type I signal peptidase SpsB, and this is then secreted extracellularly (Saenz et al. 2000; Tan et al. 2018). AgrC is a membrane receptor kinase that can be phosphorylated after binding with AIP and activate the regulatory protein AgrA, which regulates the expression of a range of downstream genes by binding the promoters P2 and P3 (Wang and Muir 2016). The RNAII transcription box regulated by promoter P2 includes the *agr* operons of the *agrA*, *agrB*, *agrC*, and *agrD* genes, while promoter P3 activates the RNAIII transcription box, expressing effector genes that encode virulence factors and biofilm proteins (Ji et al. 1995; Mayville et al. 1999). In general, the production of *agr* components increases in an autocatalytic way (Tan et al. 2018). Extracellular AIP is recognized by the two-component system (TCS) to produce more AIPs and regulate the expression of downstream genes expression, such as those related to virulence and biofilm formation. Therefore, in the absence of antibiotics, inhibitors of the *agr* system may have antibacterial effects. Consequently, most studies in AIP system focus directly on inhibitors of signaling molecules rather than on their combination with antibiotics. The *agr* system is a positively regulated auto-loop system; therefore, any step of the circuit can be a target for inhibitors to disturb AIP production. The targets of AIP system inhibitors mainly include the type I signal peptidase SpsB, the regulatory protein AgrA, and the membrane receptor kinase AgrC (Martinez et al. 2019). In Gram-positive bacteria, the leader and tail segments need to be removed from the precursor peptide to generate a complete AIP signal. The inhibitor P + 1 can inhibit the activity of the signal peptidase SpsB in a dose-dependent manner without affecting the growth of *S. aureus*, thereby reducing the synthesis of AIP signals and inhibiting the signaling system while reducing biofilm formation and *β*-lactamase production to increase sensitivity to antibiotics (Buzder-Lantos et al. 2009; Kavanaugh et al. 2007). NIF is also a SpsB inhibitor with better stability and stronger inhibition than P + 1 (Kavanaugh et al. 2007). The compound *ω*-hydroxyemodin (OHM) produced by *Penicillium* inhibits the *agr* system of *S. aureus* by interfering with the binding of AgrA to the downstream promoter P2 (Daly et al. 2015). AgrA inhibitors also include other compounds, such as savirin, naphthalene derivatives and biaryl compounds, which can bind to the C- or N-terminal of AgrA to prevent it from binding to DNA (Khodaverdian et al. 2013; Martinez et al. 2019; Sully et al. 2014). Finally, AIP analogs can bind to receptor proteins antagonistically, thereby inhibiting signaling communication. For example, AIP-III D4A is more stable than AIP-III and can inhibit the activity of the AgrC receptor protein of the signaling pathway in *S. aureus* (Tal-Gan et al. 2016).

The relationship between the AIP system and antibiotic resistance has been less studied than those of other signaling systems, but there is evidence that it can potentially regulate bacterial antibiotic resistance. Analysis of a large number of *agr* deletion mutant strains by Coelho et al. (2008) indicated that RNAIII effectors can regulate the formation and accumulation of biofilms. In *Streptococcus mutans*, there exists a competence-stimulating peptide (CSP)-mediated signaling system (Li et al. 2001). It was found that a mutant strain lacking the signal peptide synthesis gene *comC* formed a loosely structured biofilm, while a mutant strain lacking the sensor and response protein-encoding gene *comD* formed a biofilm with reduced biomass, revealing that the signaling system of this species is closely related to biofilm formation (Li et al. 2002). The microbial resistance caused by biofilms has been significantly increased, and approximately 65% of human infections were associated with biofilms (Mukherjee et al. 2006). Biofilm formation is one of the main virulence factors of *Staphylococcus*, which is related to chronic infection and antibiotic resistance (Gunther et al. 2017; Scherr et al. 2014). Thus, the AIP system may also regulate antibiotic resistance through biofilms like other signaling systems.

A large number of AIP communication inhibitors are analogs of AIP (Chen et al. 2018), which do not directly attack or inhibit bacteria and are beneficial for delaying the development of antibiotic resistance. Therefore, in future, it should be possible to search for adjuvants used in combination with antibiotics on the basis of the AIP system to treat Gram-positive antibiotic-resistant bacterial infections. For example, the small-molecule antiviral drug F19, an AgrA inhibitor, blocks the binding of the staphylococcal transcription factor AgrA and its promoter and enhances the activity of *β*-lactam and fluoroquinolone antibiotics. F19 alone or in combination with antibiotics can prevent and treat the infection of Gram-positive pathogens (Greenberg et al. 2018).

## Indole

**Fig. 5:**
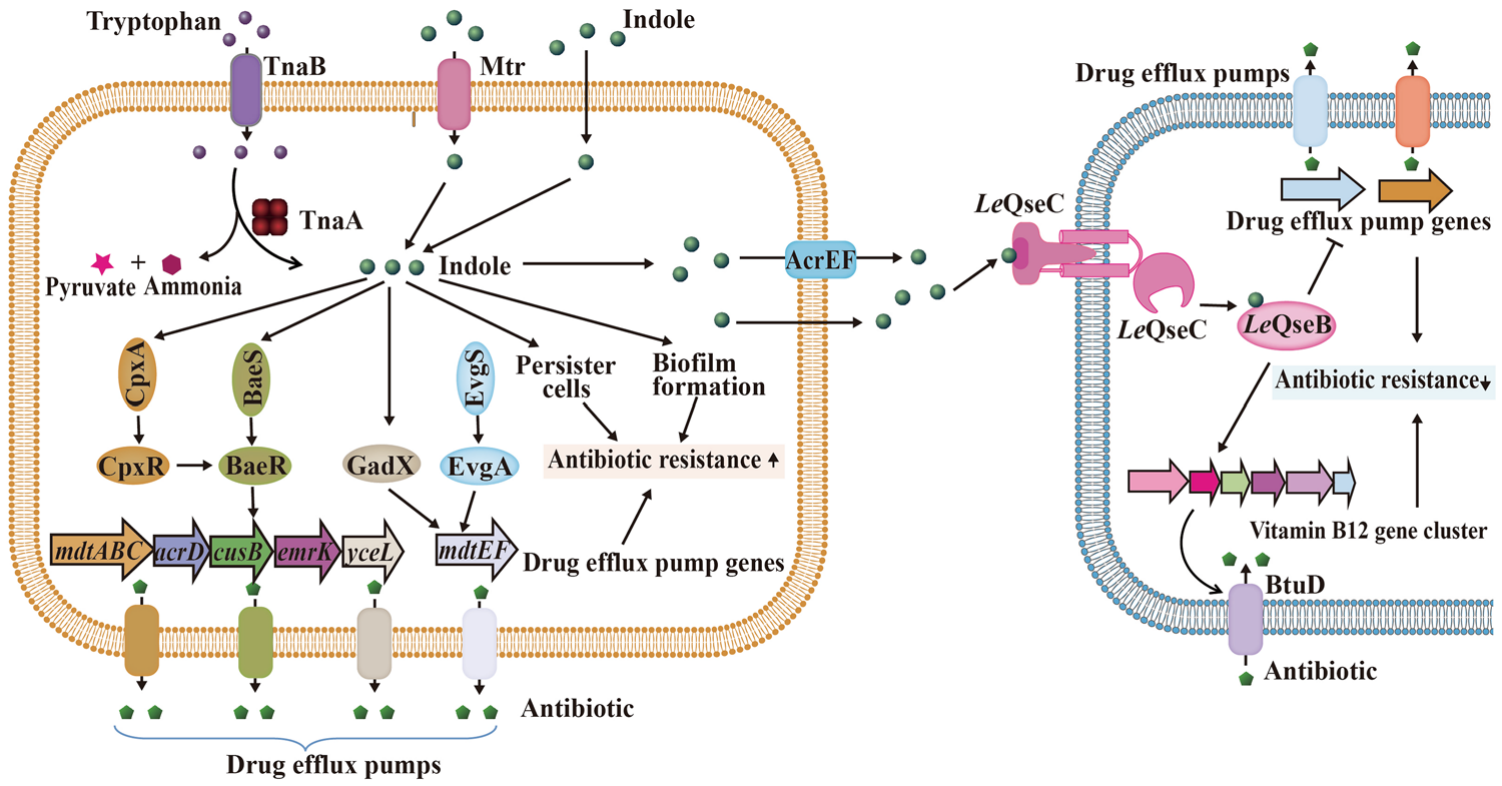
The indole-mediated signaling system. The left cell shows the mechanism by which indole enhances antibiotic resistance, and the right cell shows the mechanism by which indole reduces antibiotic resistance

Indole (Fig. 5) is a novel interspecific signaling molecule that exists widely in nature and is produced by bacteria. The mechanism by which indole is synthesized in *E. coli* has been studied in detail. The tryptophanase (TnaA), Mtr and AcrEF proteins participate in indole synthesis and transportation (Snell 1975; Wang et al. 2001; Yanofsky et al. 1991). As a signal molecule, indole can mediate complex intraspecific and interspecific communication among bacteria and their hosts (Zarkan et al. 2020), and marine fungi are important sources of indole derivatives (Cao and Wang 2020; Meng et al. 2021). Indole controls various physiological functions of microorganisms, such as bioluminescence, plasmid stability, cell growth and division, tolerance to high temperature and acid pressure, antibiotic resistance, and biofilm formation (Chant and Summers 2007; Gaimster et al. 2014; Hirakawa et al. 2005; Kuczynska-Wisnik et al. 2010; Lee et al. 2007; Nishino et al. 2008). A recent study has shown that indole can enhance the genetic diversity of aging colonies and serve as an important regulator of their resistance to complex stressful environments (Saint-Ruf et al. 2014). However, due to the complexity of the indole signaling pathway and the differences in methods, there remains a poor understanding of the function of the indole signaling pathway (Zarkan et al. 2020).

In some bacteria, indole increases antibiotic resistance. This phenomenon occurs with both endogenous and exogenous indole. Endogenous indole in *E. coli* increased antibiotic resistance by regulating the efflux pump. Examples include enhancing the expression of the *acrD* and *mdtA* multidrug efflux pump genes directly through the BaeSR/CpxAR signal regulation system (Hirakawa et al. 2005), promoting the expression of the *mdtEF* multidrug efflux pump gene through the transcription activator GadX (Hirakawa et al. 2005; Nishino et al. 2008), and regulating the AcrAB multidrug efflux pump through SdiA (Lee et al. 2007; Rahmati et al. 2002). Moreover, endogenous indole is also involved in the formation of bacterial biofilms (Domka et al. 2006). Deletion of the TnaA gene reduced the biofilm formation ability of *E. coli* S17-1, which was restored to its original level after indole was added (Wang et al. 2001).

Exogenous indole also regulates efflux pumps and biofilms of non-indole producing bacteria. *S. typhimurium* does not produce indole, but indole can regulate the expression of its *acrAB* multidrug efflux pump gene through the transcriptional regulatory factor RamAR (Nikaido et al. 2008, 2011). Similarly, Molina-Santiago et al. (2014) found that indole can promote the expression of a multidrug efflux pump TtgGHI in *Pseudomonas putida*, strains that do not produce indole, and enhance antibiotic resistance of ampicillin. In *V. cholerae*, exogenous indole can restore biofilm formation by binding directly to the RNA synthase regulatory protein DskA and the vibrio polysaccharide (VPS) regulator VpsR to activate the expression of VPS-related genes (Mueller et al. 2007, 2009). Exogenous tryptophan and indole can also promote biofilm formation of the periodontal pathogen *Fusobacterium nucleatum* (Sasaki-Imamura et al. 2010).

In addition, indole increases antibiotic resistance by inducing the formation of persisters. The remarkable feature of persisters is that they exist in a dormant state without cell division and growth under adverse conditions, such as in the presence of antibiotics, reducing metabolic activities to maintain their own survival. However, when antibiotics or other adverse conditions disappear, persisters can immediately resume growth and cell division (Balaban 2011; Zhang 2014). Vega et al. (2012) found that under ofloxacin pressure, the survival rate of wild-type *E. coli* was significantly higher than that of a *tnaA* gene mutant strain; when exogenous indole was added, the survival rate of the *tnaA* gene mutant strain improved by an order of magnitude compared with that without the addition of indole. These phenomena suggest that indole plays a key role in inducing the formation of persisters to develop resistance to antibiotics (Vega et al. 2012).

In contrast to other reported bacteria, *Lysobacter* species showed different reactions to indole. Indole could decrease the antibiotic resistance of *Lysobacter* species, which are a new potential source of antibiotics and have intrinsic resistance to many antibiotics. Han et al. (2017) found that genetic inactivation of the TCS *Le-qseC/Le-qseB* resulted in an increase in indole production. After treatment with indole, *Lysobacter enzymogenes* becomes sensitive to antibiotics. Moreover, regardless of whether indole was used, the *Le-qseC* deletion mutant strain did not grow in medium containing ampicillin, kanamycin or gentamicin, indicating that the *Le-*QseC sensor is involved in the regulation of antibiotic sensitivity. In contrast, in the absence of indole, the *Le-qseB* deletion mutant strain grew slowly in medium containing antibiotics. However, in the presence of indole, the *Le-qseB* deletion mutant strain was highly sensitive to antibiotics. This result indicates that indole enhances the sensitivity of *L. enzymogenes* to antibiotics through the TCS *Le-qseC/Le-qseB* (Han et al. 2017). Recently, Wang et al. (2019c) indicated that exogenous indole can inhibit antibiotic resistance by regulating the expression of a novel importer. In *L. enzymogenes*, exogenous indole is recognized and transported into cells by the *Le*-QseC protein and then binds to the *Le*-QseB protein to regulate the expression of the BtuD importer. High expression of BtuD importer increases the antibiotic transport efficiency and changes the sensitivity to antibiotics. When indole and antibiotics are used together, a small amount of antibiotics can achieve bactericidal effects, dramatically reducing the dosage of antibiotics and delaying the development of antibiotic resistance (Wang et al. 2019c).

Combined applications with signaling molecules to achieve bactericidal effects have also been studied with other antimicrobial drugs, in addition to antibiotics. The study of Wang et al. (2019c) found that antimicrobial peptides combined with signaling molecules could significantly enhance the bactericidal effect. LED209, a QseC/B inhibitor, is a competitive signaling molecule of indole and can inactivate the QseC/B TCS but has no germicidal effect (Han et al. 2017). Tachyplesin I (TPI) is a cationic *β*-hairpin antimicrobial peptide that has broad-spectrum antimicrobial activity and a low MIC and has good therapeutic potential (Ohta et al. 1992). TPAD is the all-D-amino acid analog of TPI, and the antibacterial activity of TPAD is similar to that of TPI, but it exhibits significantly improved anti-enzymatic degradation stability and reduced hemolytic activity (Yu et al. 2020). However, similar to antibiotics, in addition to its bactericidal effect, TPAD causes the development of bacterial resistance through the QseC/B TCS. The combined application of TPAD and the signaling molecule LED209 significantly enhanced the bactericidal effect against pathogenic bacteria with QseC/B, such as *Stenotrophomonas* and *Pseudoalteromonas* (Yu et al. 2020).

In summary, the enhancing effect of indole on the antibiotic resistance of *E. coli*, *P. putida*, and other bacteria makes the indole signaling system a potential target for resistance inhibition. In *Lysobacter* species, indole can directly inhibit antibiotic resistance and be used as adjuvants of antimicrobial drugs. Some kinds of indole derivatives found in marine environment have antibacterial activity (Chen et al. 2020; Yang et al. 2021). The bactericidal effect of combined application of TPAD and LED209 also supports the feasibility of developing antibiotics based on indole signaling system.

## Diffusible signal factor (DSF)

DSF is another common signaling molecule of Gram-negative bacteria. It is widely present in *B. cenocepacia* (Deng et al. 2011), *Xylella fastidiosa* (Chatterjee et al. 2008), *L. enzymogenes* (Qian et al. 2013b), and other bacteria (Ryan and Dow 2011). Slater et al. (2000) found three genes closely related to the DSF signaling system, namely, the *rpfF* gene, encoding DSF synthesis, and the *rpfC* and *rpfG* genes, which are related to DSF signal transduction. All DSF family signals have a fatty acid carbon chain, and their differences are mainly reflected in the chain length, double-bond configuration, and side chain (Deng et al. 2011).

**Fig. 6:**
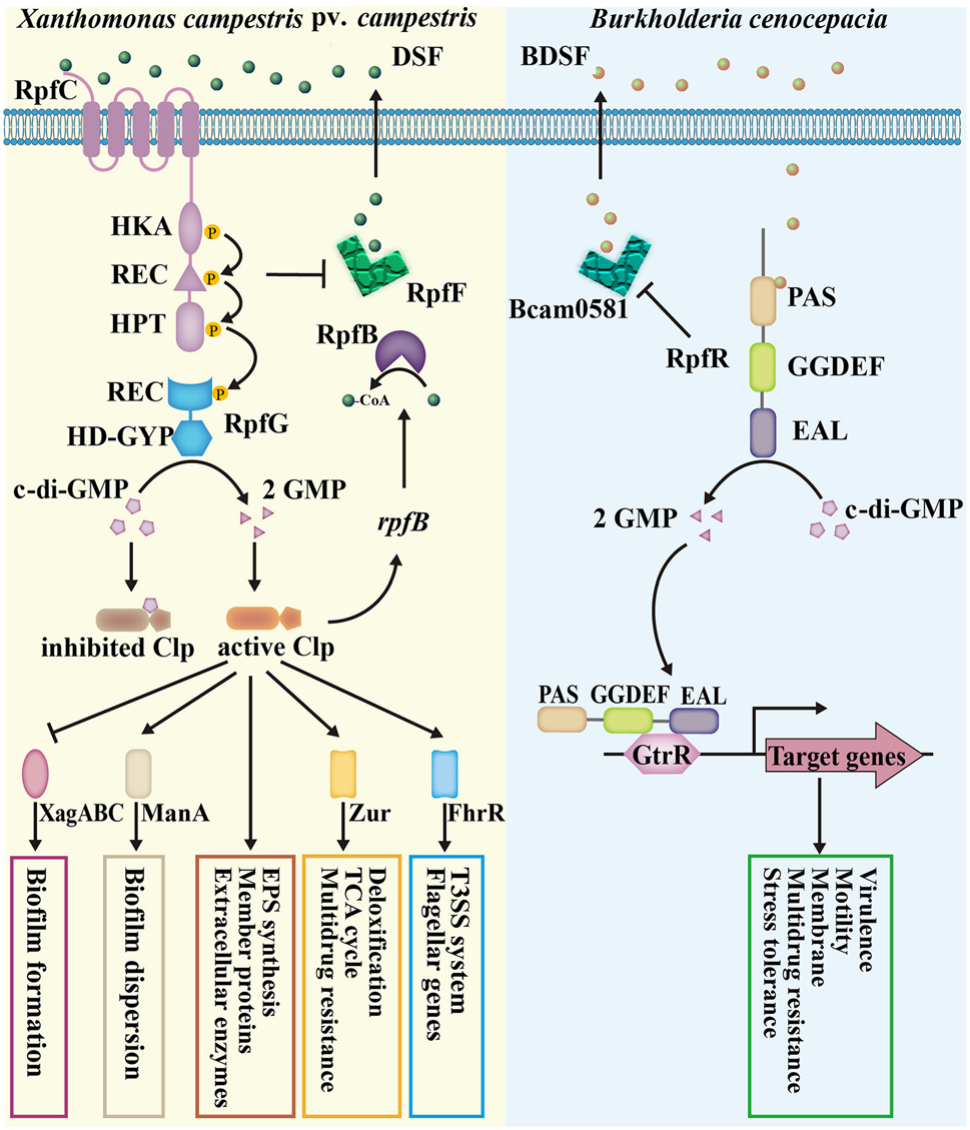
Different DSF-mediated signaling systems in *Xanthomonas campestris* pv. *campestris* and *Burkholderia cenocepacia*

In the most thoroughly studied strain, *Xanthomonas campestris* pv. *campestris* (*Xcc*), the basic framework of the DSF signaling pathway and regulatory network has been established (Fig. 6). At low cell density, the synthase RpfF binds to the DSF receptor RpfC, producing only a small number of DSF signals. When the bacterial cell density reaches a certain value, the receptor protein RpfC senses DSF accumulation outside the cell and phosphorylates itself, causing a change in the conformation of RpfC, which releases RpfF to rapidly synthesize DSF (Deng et al. 2011). At a high concentration of DSF, RpfC remains in an active state and phosphorylates RpfG to activate its phosphodiesterase, thereby degrading c-di-GMP, which is an inhibitor of the global transcription factor crp-like protein (Clp), to release and activate Clp (He et al. 2007). Clp can directly regulate the transcription of a series of target genes or indirectly regulate the expression of other genes through the downstream transcription factors FhrR and Zur to adapt to the environment (Qian et al. 2013a; Ryan et al. 2015a).

The DSF signaling system of *B. cenocepacia* has a different inner mechanism from that of *Xcc* (Fig. 6). *B. cenocepacia* has the RpfF homolog Bcam0581, which is responsible for the synthesis of BDSF, but lacks homologs of RpfC and RpfG (Boon et al. 2008). Subsequent studies have shown that RpfR, a novel receptor protein for BDSF signaling, performs the dual functions of signal sensing and signal transduction (combining the roles of RpfC and RpfG) (Deng et al. 2012). The RpfR protein contains three functional domains: PAS, GGDEF and EAL. The PAS domain senses and binds to BDSF signals, while the GGDEF and EAL domains catalyze the synthesis and degradation of c-di-GMP, respectively (Deng et al. 2012). When the accumulated BDSF signal is sensed by the PAS functional domain, a structural change occurs in RpfR that stimulates the enzyme activity of the EAL domain to degrade c-di-GMP and then regulates the expression of downstream target genes (Deng et al. 2012).

The regulation of antibiotic resistance by the DSF signaling system is a complicated process. In some bacteria, the DSF system can enhance antibiotic resistance. DSF signals of *Stenotrophomonas maltophilia* (*Stm*) positively regulate biofilm formation, virulence factors and antibiotic resistance (Fouhy et al. 2007). The relevant gene of the DSF signaling system in *Stm* is *rpfF*, and deletion of this gene significantly reduced motility, extracellular protease production, biofilm formation and *β*-lactamase production and even can lead to a significant reduction in the MICs of ampicillin (512-fold) and meropenem (267-fold) (Alcaraz et al. 2019). In addition to their roles in intraspecific signaling, signals of the DSF family also play an important role in interspecific communication (Boon et al. 2008; Deng et al. 2011). Ryan et al. (2008) found that DSF signaling molecules from *Stm* changed the biofilm structure of *P. aeruginosa* PAO1 by binding the RpfC-like receptor protein PA1396 and improved the resistance to polymyxin B. Clinical studies have shown that patients with cystic fibrosis (CF) are often infected with multiple pathogens, including *P. aeruginosa*, *B. cenocepacia* and *Stm* (Twomey et al. 2012). In the sputum of these CF patients, DSF signals produced by *B. cenocepacia* and *Stm* were found to promote *P. aeruginosa* survival and antibiotic resistance depended on PA1396 (Twomey et al. 2012). In contrast to AHL signaling molecules that positively regulate the formation of bacterial biofilms, most DSF signals are involved in regulating the degradation of biofilms. In *Xcc*, DSF attenuates biofilms by inducing the biosynthesis of biofilm-degrading enzyme *β*-1,4-mannanase (ManA) and negatively regulating the biofilm synthesis gene *xagABC* (Tao et al. 2010). Similarly, the DSF family signal produced by *P. aeruginosa* not only promotes the degradation of its own biofilms but also induces the degradation of *E. coli*, *Klebsiella pneumoniae*, *Bacillus subtilis*, and *S. aureus* biofilms (Davies and Marques 2009). In addition to biofilms, DSF also exhibited an inhibitory effect on bacterial resistance. Deng et al. (2014) found that DSF signals change the antibiotic resistance of *Bacillus cereus* through various effects, for example, by reducing the expression of efflux pump genes, inhibiting the formation of biofilms, and reducing the persistence of bacteria. In particular, T14-DSF and C15-DSF increased the sensitivity of *B. cereus* to gentamicin by 128-fold and were potential adjuvants of gentamicin (Deng et al. 2014).

The function of DSF in regulating resistance of different strains was not consistent. Even for the same species, *P. aeruginosa*, studies have reported contrasting conclusions, such as the studies between Twomey et al. (2012) and Deng et al. (2014). Why DSF functions differently in the same species remains unknown but may be due to the following: (1) The physiological behavior of different strains is different. Two strains, PAO1 (wild-type; Twomey et al.) and PA14 (clinical isolate; Deng et al.), were used in the two different studies. In addition, the genome of PA14 was 0.31 Mb larger than that of PAO1 (obtained from https://www.ncbi.nlm.nih.gov/), and PA14 was more virulent than PAO1 (Lee et al. 2006). (2) The type of antibiotics are different. The antibiotic used in the resistance enhancement experiment was polymyxin B, which reduces the stability of LPS and destroys the integrity of the outer membrane (Moffatt et al. 2019). On the other hand, the aminoglycoside antibiotics kanamycin and gentamicin were used in the resistance inhibition experiment. Aminoglycoside antibiotics act by binding to site A on the 16S rRNA decoding region of the 30S ribosome subunit to inhibit protein synthesis (Krause et al. 2016). This reflects the complexity of the DSF regulatory network and the effects of the DSF on the regulation of bacterial resistance need to be evaluated in-depth.

## Cyclic di-guanosine monophosphate (c-di-GMP)

Different from other signaling molecules, c-di-GMP only acts intracellular and is one of the second messengers of intracellular information transmission. c-di-GMP often acts as a link between other kinds of signaling molecules and downstream genes. It controls a variety of cellular functions through its signaling network, such as synthesis and secretion of the extracellular polymeric substances (EPSs), flagellar motility, and synthesis of virulence factors in bacteria (Kim et al. 2018; Ryan et al. 2015b; Wang et al. 2018). In addition, c-di-GMP can also participate in regulating the bacterial production of antibiotics. The team of Professor Qian (Chen et al. 2017; Xu et al. 2018, 2021) found that c-di-GMP is an inhibitor of the synthesis of antifungal antibiotic Heat-Stable Antifungal Factor (HSAF). c-di-GMP and its receptors, transcription factor Clp and degradation enzyme LchP, co-regulate HSAF synthesis through specific interactions in *L. Enzymogenes*.

The synthesis and degradation of c-di-GMP are determined by the combination of diguanylate cyclases (DGCs) and phosphodiesterases (PDEs) (Christen et al. 2006). The functional domain of DGCs is GGDEF, the activity of which is regulated by feedback inhibition. When c-di-GMP binds to site i in the GGDEF domain, DGCs cannot form a dimer with catalytic activity (De et al. 2008; Wassmann et al. 2007). PDEs have either an EAL or an HD-GYP domain, degrading c-di-GMP into linear pGpG or two molecules of GMP, respectively (Povolotsky and Hengge 2012; Sundriyal et al. 2014). Shang et al. (2012) revealed how c-di-GMP can be recognized by STING protein, a direct immunosensor of c-di-GMP.

Studies have shown that c-di-GMP could regulate bacterial biofilm and antibiotic resistance (Poulin and Kuperman 2021; Yi et al. 2019). For example, the presence of levofloxacin increases the intracellular c-di-GMP content of *K. pneumoniae*, resulting in increased EPS and type III fimbriae production, and finally leading to the formation of thicker biofilms to resist antibiotics (Zhang et al. 2021). c-di-GMP at light concentration will reduce EPS secretion and expression of *mrkABCDF*, which synthesizes type III fimbriae (Zhang et al. 2021). Meanwhile, the c-di-GMP signaling system is highly conserved in bacteria but does not exist in eukaryotes, making enzymes related to c-di-GMP metabolism important targets for inhibiting bacterial biofilm and antibiotic resistance. The inhibitors of c-di-GMP synthase DGC can be divided into four categories, including natural molecules, c-di-GMP analogs, GTP analogs, and small synthetic molecules, and the action modes of each type of inhibitor had been described in detail by Cho et al. (2020).

## Future directions

The release of antibiotics into the marine environment and the subsequent evolution of antibiotic resistance genes has led to increased antibiotic resistance of bacteria (Sundaramanickam et al. 2015). Meanwhile, to adapt to the environment, some bacteria in the ocean secrete metabolites that are similar to antibiotics. These metabolites can inhibit the growth of other bacteria, and stimulates them to develop stronger drug resistance. Bacterial signaling networks are closely related to antibiotic responses and resistance regulation. The strategy of suppressing antibiotic resistance through signaling systems has great potential in the treatment of bacterial infections and the marine environmental remediation. But how we are to implement this strategy is an important topic that needs in-depth study. The results will help to address the public health and ecological problems caused by bacterial resistance and antibiotics overuse. Currently, there are a series of problems to be solved. For example, the synergy mechanism between different signal networks is unclear, and the mechanism by which signaling molecules are recognized by bacterial cells is still largely unknown. In addition to biofilm formation, efflux pumps and overexpression of drug-resistant genes, other factors remain to be further studied. The functional universality of signal inhibitors needs to be further studied and increased novel signal inhibitors should be discovered. At present, studies have shown that novel marine-derived inhibitors can be obtained through new strategies such as co-culture techniques (Peng et al. 2021). Whether there are other more effective methods for inhibitor discovering is worth further investigation.
